# Draft Genome Sequence of Bifidobacterium longum subsp. *infantis* BI-G201, a Commercialization Strain

**DOI:** 10.1128/MRA.00785-20

**Published:** 2020-11-19

**Authors:** Jinan Song, Yu Xu, Di Han, Qihui Teng, Deyi Jiang, Qing Liu

**Affiliations:** a Department of Basic Research, Research & Development Center, Biogrowing Co., Ltd., Shanghai, China; Loyola University Chicago

## Abstract

Bifidobacterium longum subsp. *infantis* has been widely used in many food products such as solid beverages and dietary supplements. Here, a draft genome sequence of a commercialization strain, Bifidobacterium longum subsp. *infantis* BI-G201, is reported.

## Abstract (precis)

Bifidobacterium longum subsp. *infantis* has been widely used in many food products, such as solid beverages or dietary supplements. Here, a draft genome sequence of a commercialization strain, Bifidobacterium longum subsp. *infantis* BI-G201, was reported.

## ANNOUNCEMENT

Bifidobacterium longum subsp. *infantis* has been well recognized as a probiotic organism with many beneficial functions, such as improving gut health (1, 2), ameliorating allergic rhinitis and intermittent asthma (3), and regulating innate immunity (4). However, functional differences exist among strains, which may come from differences in their gene clusters and structures.

Bifidobacterium longum subsp. *infantis* BI-G201 was isolated from infant feces in China by our company. We adhered to the principals of the Declaration of Helsinki. The sample of infant feces was collected, timely coated onto MRS plates with 0.1% cysteine, and then cultivated at 37°C under anaerobic conditions. The isolate was picked and identified as Bifidobacterium longum subsp. *infantis* by applying a leather blue dye and performing a microscopic exam, a system biochemistry appraisal with the biochemistry appraisal reagent tube, and 16S rRNA sequence analysis.

The genomic DNA was extracted with the Omega E.Z.N.A. bacterial DNA kit, and purified genomic DNA was quantified by a TBS-380 fluorometer (Turner BioSystems, Inc., Sunnyvale, CA). High-quality DNA (optical density at 260 and 280 nm [OD_260/280_] of 1.8 to 2.0, >1 μg) was used to do further sequencing. DNA samples were sheared into 400- to 500-bp fragments using a Covaris M220 focused acoustic shearer. Illumina sequencing libraries were prepared from the sheared fragments using the NEXTflex rapid DNA sequencing (DNA-seq) kit. Briefly, 5′ ends were first end repaired and phosphorylated. Next, the 3′ ends were A tailed and ligated to sequencing adapters. The next step was to enrich the adapter-ligated products using PCR. The prepared libraries then were used for paired-end Illumina sequencing (2 × 150 bp) on an Illumina HiSeq X Ten machine. The data generated from the Illumina platform were used for bioinformatics analysis. A statistic of quality information was applied for quality trimming, by which the low-quality data (removal of adapter sequences from reads; shear removal of 5' end containing non-A, -G, -C, and -T bases; pruning the lower-quality read ends [less than Q20]; removal of reads containing 10% or higher of N bases; and discarding adapter and small fragments with a length less than 25 bp after mass pruning) can be removed to form clean data. G+C content depth distribution and k-mer frequency distribution were analyzed by SOAP software (version 2.04) to ensure the quality of reads. An assembly of the clean reads was performed using SOAPdenovo2 (version 2.04) (5). Glimmer (version 3.02) was used for coding DNA sequence (CDS) prediction (6), tRNA scan-SE (version 2.0) was used for tRNA prediction (7), and Barrnap (version 0.8) was used for rRNA prediction (8). The predicted CDSs were annotated from the nonredundant (nr), Swiss-Prot, Pfam, Gene Ontology (GO), Clusters of Orthologous Groups of proteins (COG), and KEGG databases using sequence alignment tools such as BLAST (version 2.3.0), DIAMOND (version 0.8.35) (9), and HMMER (version 3.1b2) (10). Briefly, each set of query proteins was aligned with the databases, and annotations of best-matched subjects (E value, <10^−5^) were obtained for gene annotation. The data were annotated on the free online platform Cloud Platform (Shanghai Majorbio Bio-pharm Technology Co., Ltd.).

A total of 4,905,901 × 2 raw reads and 1,481,582,102 bases were obtained from the HiSeq instrument, with the Q20 of sequencing being 98.2%. There were 57 scaffolds (67 contigs) detected in the draft genome sequence of B. longum subsp. *infantis* BI-G201, with a G+C content of 59.373%. The total length of all contigs is 2,569,356 bp, and the genome coverage is 576.64×; the largest scaffold is 292,238 bp; and the *N*_50_ and *N*_90_ values of the scaffolds are 142,829 bp and 46,699 bp, respectively. Generally, 2,281 genes, 60 tRNAs, 3 rRNA genes (including 5S, 23S, and 16S rRNA genes), 3 noncoding RNAs (ncRNAs), and 55 repeat sequences were found. There are 2,071 coding sequences, and the G+C content in the gene region is 60.66%, which is higher than that in the intergenic region (59.373%).

### Data availability.

This whole-genome shotgun project has been deposited at DDBJ/ENA/GenBank under the accession number JABNND000000000. The version described in this paper is the first version, JABNND010000000. The available raw data were released under the SRA accession number PRJNA632428.
